# 6-Methyl-1,3,5-triazine-2,4-diamine butane-1,4-diol monosolvate

**DOI:** 10.1107/S1600536812044480

**Published:** 2012-11-17

**Authors:** Rajni M. Bhardwaj, Iain Oswald, Alastair J. Florence

**Affiliations:** a Strathclyde Institute of Pharmacy and Biomedical Sciences, University of Strathclyde, 161 Cathedral Street, Glasgow G4 0RE, Scotland

## Abstract

The title co-crystal, C_4_H_7_N_5_·C_4_H_10_O_2_, crystallizes with one mol­ecule of 6-methyl-1,3,5-triazine-2,4-diamine (DMT) and one mol­ecule of butane-1,4-diol in the asymmetric unit. The DMT mol­ecules form ribbons involving centrosymmetric *R*_2_^2^(8) dimer motifs between DMT mol­ecules along the *c*-axis direction. These ribbons are further hydrogen bonded to each other through butane-1,4-diol, forming sheets parallel to (121).

## Related literature

For background to DMT and related structural studies, see: Šebenik *et al.* (1989 ▶); Kaczmarek *et al.* (2008 ▶); Portalone (2008 ▶); Xiao (2008 ▶); Fan *et al.* (2009 ▶); Qian & Huang (2010 ▶); Thanigaimani *et al.* (2010 ▶); Perpétuo & Janczak (2007 ▶); Portalone & Colapietro (2007 ▶); Delori *et al.* (2008 ▶). For details of experimental methods used, see: Florence *et al.* (2003 ▶). For ring-motif nomenclature, see: Etter (1990 ▶).
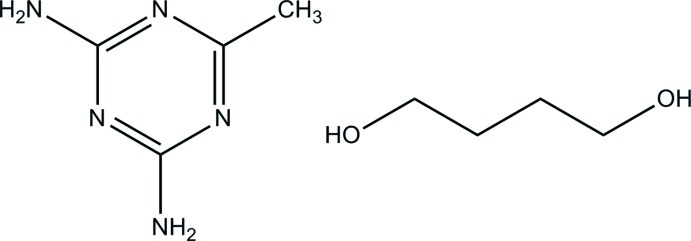


## Experimental

### 

#### Crystal data

C_4_H_7_N_5_·C_4_H_10_O_2_
*M* *_r_* = 215.27
Triclinic, 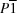

*a* = 5.8755 (3) Å
*b* = 9.0515 (5) Å
*c* = 10.7607 (5) Å
α = 87.911 (3)°
β = 74.346 (3)°
γ = 83.550 (3)°
*V* = 547.55 (5) Å^3^
*Z* = 2
Mo *K*α radiation
μ = 0.10 mm^−1^
*T* = 123 K
0.50 × 0.05 × 0.04 mm

#### Data collection

Bruker APEXII CCD diffractometer
Absorption correction: multi-scan (*SADABS*; Bruker, 2007 ▶) *T*_min_ = 0.637, *T*_max_ = 0.745
7713 measured reflections
1911 independent reflections
1288 reflections with *I* > 2σ(*I*)
*R*_int_ = 0.045

#### Refinement

*R*[*F* ^2^ > 2σ(*F* ^2^)] = 0.041
*wR*(*F* ^2^) = 0.093
*S* = 1.00
1911 reflections
155 parameters
H atoms treated by a mixture of independent and constrained refinement
Δρ_max_ = 0.20 e Å^−3^
Δρ_min_ = −0.22 e Å^−3^

### 

Data collection: *APEX2* (Bruker, 2007 ▶); cell refinement: *SAINT* (Bruker, 2007 ▶); data reduction: *SAINT*; program(s) used to solve structure: *SHELXS97* (Sheldrick, 2008 ▶); program(s) used to refine structure: *SHELXL97* (Sheldrick, 2008 ▶); molecular graphics: *Mercury* (Macrae *et al.*, 2008 ▶) and *ORTEP-3* (Farrugia, 1997 ▶); software used to prepare material for publication: *enCIFer* (Allen *et al.*, 2004 ▶) and *WinGX* (Farrugia, 1999) ▶.

## Supplementary Material

Crystal structure: contains datablock(s) I, global. DOI: 10.1107/S1600536812044480/bh2459sup1.cif

Supplementary material file. DOI: 10.1107/S1600536812044480/bh2459Isup2.mol

Structure factors: contains datablock(s) I. DOI: 10.1107/S1600536812044480/bh2459Isup3.hkl

Supplementary material file. DOI: 10.1107/S1600536812044480/bh2459Isup4.cml

Additional supplementary materials:  crystallographic information; 3D view; checkCIF report

## Figures and Tables

**Table 1:** Hydrogen-bond geometry (Å, °)

*D*—H⋯*A*	*D*—H	H⋯*A*	*D*⋯*A*	*D*—H⋯*A*
O1—H1⋯O2^i^	0.84	1.92	2.764 (2)	176
O2—H2⋯N1^ii^	0.84	1.94	2.777 (2)	178
N4—H7*N*⋯O1^iii^	0.92 (3)	2.52 (2)	3.173 (2)	128.5 (7)
N4—H8*N*⋯N2^iv^	0.85 (2)	2.19 (2)	3.037 (2)	178 (2)
N5—H9*N*⋯O1^v^	0.88 (2)	2.069 (19)	2.909 (2)	160.1 (18)
N5—H10*N*⋯N3^v^	0.87 (2)	2.14 (2)	3.008 (3)	179 (2)

Symmetry codes: (i) 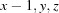; (ii) 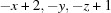; (iii) 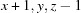; (iv) 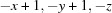; (v) 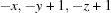.

**Fig. 1.**
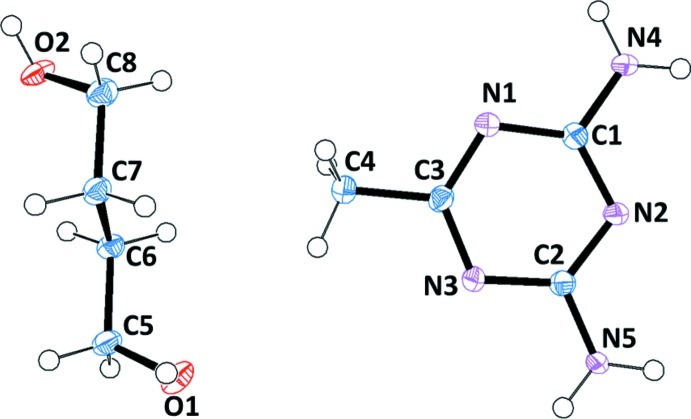
The asymmetric unit of 2,4-diamino-6-methyl-1,3,5-triazine (DMT), butane-1,4-diol solvate. Displacement ellipsoids are drawn at 50% probability level.

**Fig. 2.**
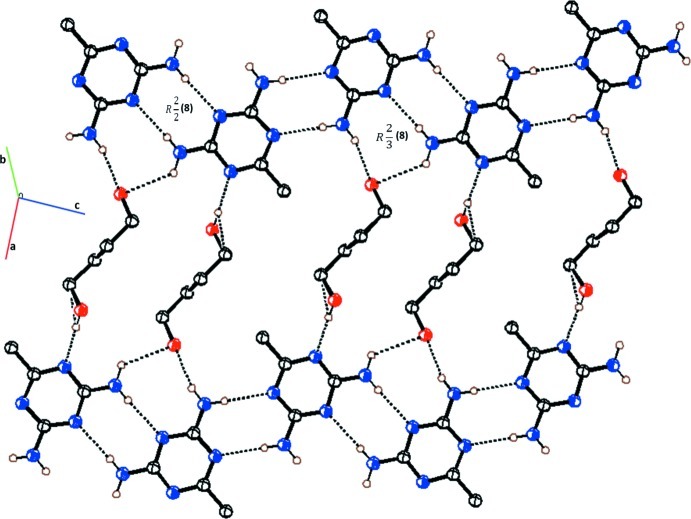
DMT molecules form ribbons through R22(8) dimer, ribbons are connected via H-bonding (shown in cyan dotted line) interactions mediated by butane-1,4-diol, thus give rise to sheet structure. C, N and H atoms are shown in black, blue and tan colour respectively. Other H atoms are omitted for clarity.

**Fig. 3.**
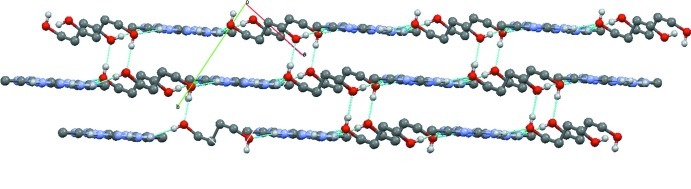
3-D Layered structure formed by sheets connected through H-bonding (cyan dotted line) mediated by butane-1,4-diol. C, N and H atoms are shown in grey, blue and white colour respectively. Other H atoms are omitted for clarity.

## supplementary crystallographic information

### Comment

2,4-diamino-6-methyl-1,3,5-triazine (DMT, acetoguanamine, Fig. 1) is used as an intermediate for pharmaceutical and resin synthesis (Šebenik *et al.*, 1989). The crystal structures of the methanol, ethanol, DMF solvates and trifluoroacetate, phthalate, nitrate and chloride salts as well as of various complexes with aliphatic dicarboxylic acids have been reported in the literature (Kaczmarek *et al.*, 2008; Portalone, 2008; Xiao, 2008; Fan *et al.*, 2009; Qian & Huang, 2010; Thanigaimani *et al.*, 2010; Portalone & Colapietro, 2007; Perpétuo & Janczak, 2007; Delori *et al.*, 2008). The sample of DMT butane-1,4-diol solvate was isolated during an experimental physical form screen. The sample was identified as a novel form using multi-sample foil transmission X-ray powder diffraction analysis (Florence *et al.*, 2003). A suitable sample for single-crystal X-ray diffraction analysis was obtained from slow evaporation of saturated butane-1,4-diol solution at room temperature. The title compound crystallizes in space group *P*1, with one molecule of DMT and one molecule of butane-1,4-diol in the asymmetric unit. Each DMT molecule forms two hydrogen-bonded dimers *via* an *R*_2_^2^(8) motif (Etter, 1990) that extends to form a ribbon structure along the *c*-direction (Fig. 2). The hydrogen bonded DMT ribbons connect to adjacent ribbons through the solvent molecule, butane-1,4-diol, thus forming a second *R*_3_^2^(8) ring motif (Fig. 2).These solvent separated ribbon structures extended to form sheets parallel to (121) plane, and are connected through hydrogen bond interactions *via* the hydroxyl groups. Solvent hydroxyl group also donates a hydrogen bond to the solvent in adjacent sheet, creating a three-dimensional layered structure (Fig. 3).

### Experimental

A single needle shape crystal was grown from the saturated solution of DMT in butane-1,4-diol by isothermal solvent evaporation at 298 K.

### Refinement

The positions of the N-bound H atoms were refined freely. All other H atoms were placed in calculated positions and refined in riding modes with *X*—H = 0.98 or 0.99 or 0.84 Å for the CH_3_, CH_2_ and OH groups, respectively. The *U*_iso_(H) values were set to 1.5 or 1.2 times *U*_eq_ of their parent C atoms for the CH_3_ and CH_2_ groups, respectively. The *U*_iso_(H) values were set to 1.5 times *U*_eq_ of their parent O atoms for the OH groups.

### Crystal data

**Table:**

C_4_H_7_N_5_·C_4_H_10_O_2_	*Z* = 2
*M**_r_* = 215.27	*F*(000) = 232
Triclinic, *P*1	*D*_x_ = 1.306 Mg m^−^^3^
Hall symbol: -P 1	Mo *K*α radiation, λ = 0.71073 Å
*a* = 5.8755 (3) Å	Cell parameters from 1602 reflections
*b* = 9.0515 (5) Å	θ = 2.3–24.6°
*c* = 10.7607 (5) Å	µ = 0.10 mm^−^^1^
α = 87.911 (3)°	*T* = 123 K
β = 74.346 (3)°	Needle, colourless
γ = 83.550 (3)°	0.50 × 0.05 × 0.04 mm
*V* = 547.55 (5) Å^3^	

### Data collection

**Table:**

Bruker APEXII CCD diffractometer	1911 independent reflections
Radiation source: fine-focus sealed tube	1288 reflections with *I* > 2σ(*I*)
Graphite monochromator	*R*_int_ = 0.045
φ and ω scans	θ_max_ = 25.0°, θ_min_ = 2.0°
Absorption correction: multi-scan (*SADABS*; Bruker, 2007)	*h* = −6→6
*T*_min_ = 0.637, *T*_max_ = 0.745	*k* = −10→10
7713 measured reflections	*l* = −12→12

### Refinement

**Table:**

Refinement on *F*^2^	Primary atom site location: structure-invariant direct methods
Least-squares matrix: full	Secondary atom site location: difference Fourier map
*R*[*F*^2^ > 2σ(*F*^2^)] = 0.041	Hydrogen site location: inferred from neighbouring sites
*wR*(*F*^2^) = 0.093	H atoms treated by a mixture of independent and constrained refinement
*S* = 1.00	*w* = 1/[σ^2^(*F*_o_^2^) + (0.0401*P*)^2^ + 0.1476*P*] where *P* = (*F*_o_^2^ + 2*F*_c_^2^)/3
1911 reflections	(Δ/σ)_max_ < 0.001
155 parameters	Δρ_max_ = 0.20 e Å^−^^3^
0 restraints	Δρ_min_ = −0.22 e Å^−^^3^
0 constraints	

### Fractional atomic coordinates and isotropic or equivalent isotropic displacement parameters (Å^2^)

**Table:**

	*x*	*y*	*z*	*U*_iso_*/*U*_eq_	
C1	0.5923 (3)	0.3881 (2)	0.16893 (18)	0.0160 (5)	
C2	0.2419 (3)	0.4825 (2)	0.30267 (18)	0.0163 (5)	
C3	0.5020 (3)	0.3277 (2)	0.38201 (19)	0.0174 (5)	
C4	0.5670 (4)	0.2448 (2)	0.49230 (19)	0.0243 (5)	
H4A	0.4317	0.2559	0.5692	0.036*	
H4B	0.7035	0.2850	0.5095	0.036*	
H4C	0.6083	0.1393	0.4705	0.036*	
C5	0.3181 (3)	0.1867 (2)	0.9133 (2)	0.0207 (5)	
H5A	0.2639	0.1078	0.9771	0.025*	
H5B	0.3114	0.2788	0.9615	0.025*	
C6	0.5730 (3)	0.1418 (2)	0.83897 (19)	0.0182 (5)	
H6A	0.6744	0.1373	0.8993	0.022*	
H6B	0.6254	0.2195	0.7737	0.022*	
C7	0.6115 (3)	−0.0072 (2)	0.77120 (19)	0.0191 (5)	
H7A	0.5611	−0.0855	0.8363	0.023*	
H7B	0.5099	−0.0033	0.7110	0.023*	
C8	0.8673 (3)	−0.0489 (2)	0.69683 (19)	0.0220 (5)	
H8A	0.8820	−0.1465	0.6551	0.026*	
H8B	0.9165	0.0260	0.6282	0.026*	
N1	0.6600 (3)	0.31119 (18)	0.26599 (15)	0.0175 (4)	
N2	0.3851 (3)	0.47401 (18)	0.18167 (15)	0.0162 (4)	
N3	0.2928 (3)	0.41094 (18)	0.40710 (15)	0.0174 (4)	
N4	0.7434 (3)	0.3769 (2)	0.05127 (17)	0.0221 (4)	
N5	0.0347 (3)	0.5670 (2)	0.32441 (19)	0.0201 (4)	
O1	0.1589 (2)	0.21134 (16)	0.83288 (14)	0.0238 (4)	
H1	0.1221	0.1293	0.8146	0.036*	
O2	1.0196 (2)	−0.05639 (16)	0.78135 (13)	0.0229 (4)	
H2	1.1154	−0.1339	0.7660	0.034*	
H7N	0.883 (4)	0.316 (3)	0.040 (2)	0.037 (7)*	
H8N	0.704 (4)	0.418 (2)	−0.013 (2)	0.028 (7)*	
H9N	−0.008 (3)	0.618 (2)	0.262 (2)	0.019 (6)*	
H10N	−0.059 (4)	0.572 (2)	0.402 (2)	0.026 (6)*	

### Atomic displacement parameters (Å^2^)

**Table:**

	*U*^11^	*U*^22^	*U*^33^	*U*^12^	*U*^13^	*U*^23^
C1	0.0178 (11)	0.0139 (11)	0.0162 (11)	−0.0025 (9)	−0.0042 (9)	0.0004 (9)
C2	0.0158 (11)	0.0155 (11)	0.0169 (11)	−0.0015 (9)	−0.0036 (9)	0.0005 (9)
C3	0.0192 (12)	0.0152 (11)	0.0180 (11)	0.0017 (9)	−0.0069 (9)	−0.0010 (9)
C4	0.0269 (12)	0.0266 (13)	0.0167 (11)	0.0075 (10)	−0.0055 (10)	0.0007 (9)
C5	0.0165 (11)	0.0232 (13)	0.0221 (11)	0.0028 (9)	−0.0062 (9)	−0.0016 (9)
C6	0.0144 (11)	0.0194 (12)	0.0210 (11)	−0.0004 (9)	−0.0055 (9)	−0.0001 (9)
C7	0.0166 (11)	0.0200 (12)	0.0212 (11)	−0.0005 (9)	−0.0067 (9)	0.0007 (9)
C8	0.0202 (12)	0.0240 (13)	0.0230 (11)	0.0021 (9)	−0.0088 (10)	−0.0032 (9)
N1	0.0176 (9)	0.0180 (10)	0.0151 (9)	0.0024 (7)	−0.0031 (8)	−0.0012 (7)
N2	0.0151 (9)	0.0164 (9)	0.0148 (9)	0.0024 (7)	−0.0016 (7)	0.0008 (7)
N3	0.0184 (9)	0.0175 (10)	0.0145 (9)	0.0025 (7)	−0.0033 (7)	0.0023 (7)
N4	0.0184 (11)	0.0272 (11)	0.0152 (10)	0.0082 (9)	0.0004 (9)	0.0015 (8)
N5	0.0172 (10)	0.0258 (11)	0.0120 (10)	0.0071 (8)	0.0005 (9)	0.0047 (8)
O1	0.0201 (8)	0.0202 (8)	0.0340 (9)	0.0013 (7)	−0.0139 (7)	0.0009 (7)
O2	0.0171 (8)	0.0223 (9)	0.0299 (9)	0.0073 (6)	−0.0102 (7)	−0.0055 (7)

### Geometric parameters (Å, º)

**Table:**

C1—N4	1.336 (2)	C6—C7	1.522 (3)
C1—N2	1.345 (2)	C6—H6A	0.9900
C1—N1	1.357 (2)	C6—H6B	0.9900
C2—N5	1.331 (3)	C7—C8	1.512 (3)
C2—N2	1.346 (2)	C7—H7A	0.9900
C2—N3	1.362 (2)	C7—H7B	0.9900
C3—N3	1.334 (2)	C8—O2	1.434 (2)
C3—N1	1.342 (2)	C8—H8A	0.9900
C3—C4	1.494 (3)	C8—H8B	0.9900
C4—H4A	0.9800	N4—H7N	0.92 (2)
C4—H4B	0.9800	N4—H8N	0.85 (2)
C4—H4C	0.9800	N5—H9N	0.87 (2)
C5—O1	1.431 (2)	N5—H10N	0.87 (2)
C5—C6	1.512 (3)	O1—H1	0.8400
C5—H5A	0.9900	O2—H2	0.8400
C5—H5B	0.9900		
			
N4—C1—N2	117.48 (18)	C7—C6—H6B	108.7
N4—C1—N1	117.24 (18)	H6A—C6—H6B	107.6
N2—C1—N1	125.28 (18)	C8—C7—C6	113.03 (17)
N5—C2—N2	118.72 (19)	C8—C7—H7A	109.0
N5—C2—N3	116.41 (18)	C6—C7—H7A	109.0
N2—C2—N3	124.86 (18)	C8—C7—H7B	109.0
N3—C3—N1	125.76 (18)	C6—C7—H7B	109.0
N3—C3—C4	117.45 (18)	H7A—C7—H7B	107.8
N1—C3—C4	116.79 (17)	O2—C8—C7	110.49 (16)
C3—C4—H4A	109.5	O2—C8—H8A	109.6
C3—C4—H4B	109.5	C7—C8—H8A	109.6
H4A—C4—H4B	109.5	O2—C8—H8B	109.6
C3—C4—H4C	109.5	C7—C8—H8B	109.6
H4A—C4—H4C	109.5	H8A—C8—H8B	108.1
H4B—C4—H4C	109.5	C3—N1—C1	114.53 (16)
O1—C5—C6	113.42 (16)	C1—N2—C2	114.71 (17)
O1—C5—H5A	108.9	C3—N3—C2	114.85 (17)
C6—C5—H5A	108.9	C1—N4—H7N	118.7 (14)
O1—C5—H5B	108.9	C1—N4—H8N	120.1 (15)
C6—C5—H5B	108.9	H7N—N4—H8N	121 (2)
H5A—C5—H5B	107.7	C2—N5—H9N	121.4 (13)
C5—C6—C7	114.11 (17)	C2—N5—H10N	119.2 (14)
C5—C6—H6A	108.7	H9N—N5—H10N	119.4 (19)
C7—C6—H6A	108.7	C5—O1—H1	109.5
C5—C6—H6B	108.7	C8—O2—H2	109.5
			
O1—C5—C6—C7	−64.5 (2)	N1—C1—N2—C2	1.1 (3)
C5—C6—C7—C8	179.55 (17)	N5—C2—N2—C1	178.99 (18)
C6—C7—C8—O2	59.0 (2)	N3—C2—N2—C1	−0.6 (3)
N3—C3—N1—C1	−0.1 (3)	N1—C3—N3—C2	0.5 (3)
C4—C3—N1—C1	179.68 (18)	C4—C3—N3—C2	−179.23 (18)
N4—C1—N1—C3	179.49 (18)	N5—C2—N3—C3	−179.76 (18)
N2—C1—N1—C3	−0.8 (3)	N2—C2—N3—C3	−0.1 (3)
N4—C1—N2—C2	−179.17 (19)		

### Hydrogen-bond geometry (Å, º)

**Table:**

*D*—H···*A*	*D*—H	H···*A*	*D*···*A*	*D*—H···*A*
O1—H1···O2^i^	0.84	1.92	2.764 (2)	176
O2—H2···N1^ii^	0.84	1.94	2.777 (2)	178
N4—H7*N*···O1^iii^	0.92 (3)	2.52 (2)	3.173 (2)	128.5 (7)
N4—H8*N*···N2^iv^	0.85 (2)	2.19 (2)	3.037 (2)	178 (2)
N5—H9*N*···O1^v^	0.88 (2)	2.069 (19)	2.909 (2)	160.1 (18)
N5—H10*N*···N3^v^	0.87 (2)	2.14 (2)	3.008 (3)	179 (2)

Symmetry codes: (i) *x*−1, *y*, *z*; (ii) −*x*+2, −*y*, −*z*+1; (iii) *x*+1, *y*, *z*−1; (iv) −*x*+1, −*y*+1, −*z*; (v) −*x*, −*y*+1, −*z*+1.
